# Towards a neural model of creative evaluation in advertising: an electrophysiological study

**DOI:** 10.1038/s41598-020-79044-0

**Published:** 2020-12-15

**Authors:** Shujin Zhou, Junlong Luo, Tingting Yu, Dan Li, Yue Yin, Xiaochen Tang

**Affiliations:** 1 Department of Psychology, Shanghai Normal University, 100 Guilin Road, Xuhui District, Shanghai, 200234 China; 2 Department of Psychology, School of Psychology and Cognitive Science, East China Normal University, 3663, North Zhong Shan Road, Shanghai, 200062 China; 3 Shanghai Mental Health Center, Shanghai Jiaotong University, 600 Wanping South Road, Xuhui District, Shanghai, 200030 China

**Keywords:** Psychology, Neuroscience, Perception, Problem solving

## Abstract

Although it is increasingly recognized that evaluation is a key phase for a two-fold creativity model, the neural model is not yet well understood. To this end, we constructed a theoretical model of creative evaluation and supported it with neural evidence through event-related potentials (ERPs) technology during a creative advertising task. Participants were required to evaluate the relationship between target words and advertising that systematically varied in novelty and usefulness. The ERPs results showed that (a) the novelty-usefulness and novelty-only conditions evoked a larger N1-P2 amplitude, reflecting an automatic attentional bias to novelty, and (b) these two novelty conditions elicited a larger N200-500 amplitude, reflecting an effort to process the novel content; (c) the novelty-usefulness and usefulness-only conditions induced a larger LPC amplitude, reflecting that valuable associations were formed through retrieval of relevant memories. These results propose a neural model of creative evaluation in advertising: the N1-P2, N200-500, and LPC should be the key indices to define three sub-processes of novelty perception, conception expansion, and value selection, respectively.

## Introduction

Creativity is generally defined as the ability to produce ideas or products that are both novel (original and unusual) and useful (appropriate and valuable)^1–4^. Paralleling such two-criterion definitions, psychological theories have suggested a dual-process model of creativity which involves generation and evaluation phases^5–8^. Where the former focuses on formulating original ideas by connecting different ideas in unusual ways^9^, the latter focuses on the value selection and eventual implementation of those ideas^10^. It appears to be particularly worth mentioning that idea evaluation has received far less attention than idea generation, indicating a necessity for additional exploration of evaluative processing^11^. With that, the main contribution of this study is to construct a theoretical model that focuses on creative evaluation and supporting it with neural evidence.

Evaluation is a key stage of the creativity process^12,13^, several subsequent studies have suggested various frameworks around this. For instance, Mumford, Lonergan, and Scott^14^ proposed a three-processes, beginning with the appraisal of ideas, by identifying the relevant standards^15^, then forecasting the potential consequences through mental simulation^16^, and finally, refining and revising ideas into valuable ones^17^. Subsequently, Zhang and Zhang^18^ suggested that creative evaluation includes three categories: novelty, value, and practicality. Recently, Kleinmintz et al.^19^ followed the definition of creativity to further divide the evaluation phase into three sub-stages: valuation (evaluation of usefulness), monitoring (evaluation of novelty), and selection (interplay between these two stages). On the whole, the framework of the creative evaluation phase was extended from the nature of creativity, namely, novelty and usefulness. The difference is that some believe that novelty should be evaluated first, while others consider usefulness first. In fact, novelty recognition is the crucial starting point for evaluation from the exogenous ideas^20^. Given the biases that arise from the judgments of an individual’s own ideas, the present study will predefine a set of ideas generated by others as the objective of evaluation^21^. By definition, evaluation is a discrete process that entails labeling the generated ideas according to their perceived novelty and usefulness, returning to the ideas for additional expansion, and eventually selecting those ideas into a form that is of value^6,22,23^. Taken together, for exogenous ideas, we proposed that the evaluative process consists of three sub-stages: (1) novelty perception, whereby one gains attentional bias to distinguish the perception of novelty and usefulness, (2) concept expansion, whereby one assesses the novelty component in a concept expansion manner, and (3) value selection, the ultimate evaluation of usefulness. Overall, although there is no lack of studies that have attempted to explore the processing of creative evaluation, the neural evidence of the model of creative evaluation remains to be further explored.

Previous studies have obtained some important findings about the specific neural networks of creative evaluation. For example, Huang, Fan, and Luo^24^ presented the neural evidence of novelty and appropriate judgments. They found that the evaluation of novelty is associated with the bilateral dorsolateral prefrontal cortex (DLPFC) and temporoparietal junction (TPJ), while the evaluation of appropriateness was associated with the orbitofrontal cortex (OFC), TPJ, posterior cingulate cortex (PCC), amygdala, ventral striatum, and hippocampus. All aforementioned studies, however, focused on the neural structure about creative evaluation with the help of fMRI technology rather than on the neurocognitive mechanisms underlying the processing of evaluation. Indeed, event-related potentials (ERPs) may provide a temporal resolution of cognitive processes^25^, and therefore is very suitable for investigating the time course of electrophysiological processes during creativity evaluation.

As already noted, novelty and usefulness are the central components of the model of creative evaluation^24^. In fact, as a creative product, creative advertisement (hereafter ad) effectively reflects novelty and usefulness^3,26^. Specifically, in an ad context, novelty refers to the incongruent and atypical elements that are depicted^27^, thereby making the ad distinct and expected to attract attention^28^. Usefulness requires an ad to be not only appropriate and meaningful but also useful^29,30^. Accordingly, based on its higher application value^29^, it is of ecological and practical importance to research the creative evaluation of advertising.

In order to demonstrate the novelty and usefulness features clearly, the present study adopted a new paradigm to investigate the processing mechanisms of creative evaluation using ERPs technology. Specifically, the participants were first presented with a target word, followed by an ad image (including a creative description). They were then asked to evaluate the relationship between the word and the ad. According to their evaluations, there were three conditions in the experiment: the novelty-usefulness (NU) condition, the usefulness-only (U) condition, and the novelty-only (N) condition. In reality, the target word in the “NU condition” was the theme of the ad, which, thus, reflected novelty and usefulness. The “U condition” pertained to information directly perceived from the ad image rather than the theme of the ad, and shows as a non-novel but useful feature. The “N condition” was unrelated to the ad. That is, the individual could not effectively connect to the ad given the lack of usefulness. Interestingly, this irrelevance, owing to the initial anticipation of the non-relevant word, is inconsistent with the ad image, and thus meets the criteria of novelty. Hence, based on the stimuli and paradigm, it is possible to allow participants to evaluate the creativity of the ads centering around the two features to reveal the processing of creative evaluation.

In the initial stage, participants would intuitively assess, in habitual cognitive modes, whether the word was deviant from the ad, as thus, the NU and N conditions were bound to lead to more attentional bias due to the detection of intuitive irrelevance in the word-ad. After detecting the deviant information, participants would attempt to establish a new semantic connection through the expansion of the existing concepts. Eventually, participants would select ideas according to the different levels of associated appropriateness of the conceptual combinations. Based on the previously reviewed neuroscientific findings, we hypothesized that: (1) The early ERP components, such as the frontal N1 and fronto-central P2 associated with attentional allocation^31,32^, may contribute to novelty perception stage that would enable the NU and N conditions to elicit more N1/P2-like responses in contrast to the U condition^33,34^. (2) And N400 component is particularly associated with processing semantic deviance^35,36^, thus, relative to U condition, it seems reasonable to predict that the NU and N conditions will elicit N400-like effect that was found to be limited to centroparietal regions in the concept expansion stage^26,37^. (3) In the value selection stage, the selection of valuable ideas requires the recall of relevant knowledge, thus, the NU and U conditions will elicit the activation associated with the memory recollection (e.g., LPC) compared to N condition^38,39^.

## Results

### Behavioral performance

Only those trials where the participants pressed the “1” key in the N condition, the “2” key in the U condition, and the “3” key in the NU condition were included in the analyses. For the correct ratio, one-way (three level: NU, N, U) repeated ANOVA measures revealed the significant main effect for the condition, *F* (2, 46) = 19.61; *p* < 0.001; η_p_^2^ = 0.46 > 0.14. The results indicated that the correct ratio of the N condition (*M* = 0.96, *SD* = 0.04) was significantly higher than those of the NU condition (*M* = 0.73, *SD* = 0.19, *p* < 0.001) and the U condition (M = 0.81, SD = 0.18, *p* < 0.001). However, no significant difference was observed between NU and U conditions (*p* > 0.05). In terms of mean reaction times (RTs), the main effect of the condition was statistically significant: *F* (2, 46) = 13.29; *p* < 0.001; η_p_^2^ = 0.37 > 0.14. The RTs were significantly longer under the NU condition (*M* = 2,124.51 ms, *SD* = 545.11) than under the N condition (*M* = 1,749.37 ms, *SD* = 392.18, *p* < 0.001) and U condition (*M* = 1,800.11 ms, *SD* = 636.39, *p* = 0.002). No significant difference was observed between N and U conditions (*p* > 0.05).

### ERP results

N1: The repeated ANOVA measures showed significant main effects for the condition: *F* (2, 46) = 6.90, *p* = 0.002, η_p_^2^ = 0.23 > 0.14. Bonferroni corrected pairwise comparisons demonstrated that the NU condition (*p* = 0.007) and N condition (*p* = 0.022) elicited a significantly greater N1 mean amplitude than the U condition. There was also no significant difference between NU and N conditions (*p* > 0.05). Furthermore, the main effect of electrode and the difference between the NU and N conditions were not significant.

P2: Significant main effects were observed for the condition: *F (*2, 46*)* = 9.94*, p* < 0.001, η_p_^2^ = 0.30 > 0.14; and the electrode: *F* (14, 322) = 7.48, *p* < 0.001, η_p_^2^ = 0.26 > 0.14. The pairwise comparisons revealed that the P2 mean amplitude induced by the U condition was significantly greater than that of the NU (*p* = 0.001) and N (*p* = 0.005) conditions. Furthermore, the P2 in the NU condition did not differ significantly from that of the N condition (*p* > 0.05). There was also no significant interaction between condition and electrode.

N200-500: the results revealed a significant main effect of condition: *F* (2, 46) = 17.10, *p* < 0.001, η_p_^2^ = 0.43 > 0.14, indicating that the NU (*p* = 0.001) and N conditions (*p* < 0.001) elicited the larger N200-500 amplitudes than U condition. While the NU and N conditions did not differ significantly (*p* > 0.05). Moreover, the main effects were observed for the electrode: *F* (20, 460) = 49.00, *p* < 0.001, η_p_^2^ = 0.68 > 0.14. There was an interaction between condition and electrode, *F* (40, 920) = 4.73, *p* < 0.001, η_p_^2^ = 0.17 > 0.14. To analyze this interaction, a simple-effect analysis was conducted. The results suggest that NU condition evoked larger N200-500 amplitudes than U condition at most of electrodes excluding FP2, P3, PO3, POZ, and PO4 (all *p* < 0.05), and N condition evoked an increased N2 than the U condition at the most of electrodes excluding PO3 (all *p* < 0.05).

LPC: The ANOVA revealed the significant main effects for the condition: *F* (2, 46) = 7.41, *p* = 0.002, η_p_^2^ = 0.24 > 0.14, and the electrode: *F* (20, 460) = 14.54, *p* < 0.001, η_p_^2^ = 0.39 > 0.14. The pairwise comparison showed that the LPC mean amplitude was more positive for the NU condition (*p* = 0.026) and U condition (*p* = 0.007) than for the N condition. However, the NU and U conditions did not differ significantly (*p* > 0.05). In addition, we found a significant interaction between the condition and electrode: *F* (40, 920) = 5.61, *p* < 0.001, η_p_^2^ = 0.20 > 0.14. The simple effect analysis found that the difference between the NU and N conditions was significant at the following sites, FPZ, FP2, FZ, F4, FCZ, FC4, CZ, and C4 (all *p* < 0.05); the marked difference between U condition and N condition at the FP1, FPZ, FP2, FZ, F4, FC3, FCZ, FC4, CZ, C4, CPZ, and PZ (all *p* < 0.05).

## Discussion

In this study, ERPs are utilized to measure the electrophysiological and evaluative responses to creative advertising. It is the first ERP study to explore electrophysiological evidence of the model of creative evaluation in advertising. The RT to the NU condition was the longest and the correct ratio was the lowest among the three conditions, though there was no significant difference for the correct ratio between the NU and U conditions, indicating that individuals spent more time evaluating the creative ad for a better evaluation. Combined, the results imply that the processing of creative evaluation is the most complicated and requires more cognitive resources to evaluate novelty and usefulness features simultaneously. More importantly, analysis of ERPs revealed that the evaluation of creative ads elicited different waves in comparison with non-novel (i.e., U) ads before the 500 ms time window and elicited different waves compared to useless ads after the 500 ms window. These ERP waves may reflect the different activities involved in the three sub-processes of creative evaluation.

Consistent with the hypothesis of a privileged perception of the novelty feature, the two novelty (NU and N) conditions evoked a more negative N1 at the frontal brain scalp and a more positive P2 with a frontocentral topography than the non-novel condition in the novelty perception stage. Frontal N1 and frontocentral P2 were associated with the early stage of visual processing^33,40–42^; furthermore, their amplitudes reflected attention tendency of the two novelty conditions and difficulty in processing visual selective attention^43,44^. The two concepts are frequently described as N1–P2 complex components^44–46^, reflecting, thus, a relatively low-level, rapid, and automated process^33^. The attentional bias occurred when the ad image (e.g., depicting a tower of cards) was incongruous with the initial expectation primed by the target word in the two novelty conditions (e.g., “express” for the NU condition and “razor” for the N condition), allowing individuals to distinguish novelty from usefulness in an early stage of visual perception. However, in the U condition, the participants were prone to having logically consistent anticipation given the coherence between the target word (e.g., “cards” for the U condition) and the ad image. Thus, the two novelty conditions induced more negative N1–P2, which might indicate that the initial evaluation of the perceived novelty feature requires additional attention and resources from individuals to prepare for the further process of novelty evaluation subsequently.

Once novel conditions attract increased attention from individuals, then components that are associated with the increased effort to resolve cognitive conflict may be elicited in the concept expansion stage. The results clearly demonstrate that the word-ad relationships participants found to be irrelevant elicited significantly higher N200-500 amplitudes in fronto-centro-parietal sites than those evaluated as relevant to the image (U condition). Moreover, the N200-500 amplitude difference between the N condition and NU condition was not significant, which indicates that the N200-500 is more sensitive to the levels of novelty but not to the usefulness in a creative ad. As predicted, the N200-500 elicited by the novelty feature fits perfectly with the N400, involving an effort toward the processing of semantic information that is incongruent with semantic expectancy^36^. These findings almost fit with the study on metaphor processing by Rutter et al*.*^37^. The greater N400 (time window 300–500 ms) amplitudes for the novel metaphoric (rated as highly novel and highly useful) and nonsensical phrases (rated as highly novel and less useful) compared to literal phrases (rated as less novel and highly useful), the greater effort to retrieve the concepts and their distinct features. We can thus conclude that the N200-500 mirrors the effort of the cognitive conflict in semantically incongruent contexts. Specifically, for the U condition, the individuals followed the ad content automatically and effortlessly because the target word (e.g., cards) is the information in the image (e.g., depicting a tower of cards) and represents an established link between strongly associated concepts. In comparison, the two novelty conditions require more effort to break habitual cognitive modes and establish connections between two previously unrelated concepts conveyed through the word and ad (e.g. NU condition: “express” and cards; N condition: “razor” and cards). It should be noted that the conflicts caused in the two novelty conditions are not completely homogeneous: the inability to successfully integrate the conflict in the N condition, while that in the NU condition is possible to be resolved into a novel conceptual combination. Even though the ERP results indicate that the difference in N200-500 induced by the two novelty conditions is not significant, indicating that irrespective of the kind of conflict that emerges, the effort exerted in attempting to resolve it is the same.

After an attempt to bring the two weakly related concepts (word-ad) into a relation, it is only through the retrieval of the usefulness information that enables valuable semantic connections to be forged and integrated within the semantic memory systems^37^. Thus, the two usefulness (NU and U) conditions, in contrast to the useless (i.e., N) condition, evoked a larger LPC with a frontocentral topography during the value selection stage. Previous studies have revealed that the LPC was responsible for the memory recollection^38^ and the elaborate processing of the input information based on long-term memory^39,47^; the dissociation between the NU and N conditions seems reasonable because forming the word and ad into a valuable combination without the useful and relevant memory is not feasible for participants. In fact, the value selection stage requires the recruitment of the relevant memory system to form valuable associations to navigate future decisions. Specifically, in the NU condition, the established semantic connections can be refined into valuable associations by retrieving the relevant semantic memory, e.g. the refined usefulness information is that the stability of the card tower conveyed via the ad highlights the quality assurance of express delivery. Furthermore, for the U condition, or usefulness information can be recalled through a much shallower processing without the need to elaborate deeper on semantic knowledge, e.g. participants soon realized that the “cards” are only the main information of the ad image, and thus judged this association as relevant to the image. Therefore, the LPC effect reflects the selection of a valuable manner in which the concepts can be linked with each other, regardless of the usefulness of the novelty–usefulness or usefulness-only condition. Moreover, the LPC effect is the concentrated embodiment of the evaluation of the usefulness feature.

To summarize, we found that different stages of the creative evaluation process have different behavioral and electrophysiological responses across different brain regions in different time windows, and further proposed the neural model of the creative evaluation process. The novelty perception stage begins early in the frontal N1 and frontocentral P2, reflecting the automatic attentional tendency to the novelty feature. The stage of concept expansion is then linked to fronto-centro-parietal N200-500 component, reflecting controlled cognitive processes on the effort to resolve the deviant semantic. The last stage of creative evaluation, the value selection, shows a strong LPC effect at the frontocentral sites, reflecting the forming of valuable associations by retrieving the relevant memory. Collectively, these findings imply that creative evaluation is a complex process that involves automated and controlled processing. In addition, N1-P2, N200-500, and LPC can be proposed as indices relevant to the processes of creative evaluation. Intriguingly, the ERP effects of the evaluation phase of creativity in the present study were relatively similar to the generative phase of creativity in existing researches, such as: N1-P2 for Xing et al*.*^46^, N380 in the 250–500 ms for Mai et al.^48^ and LPC in the 620–800 ms for Shen et al*.*^49^. It therefore appears that the evaluative process itself may incorporate the generative process, whereas ideas are reformed to ensure their successful implementation^50^. In other words, the generation and evaluation phases are inseparable and as the two-fold model of creativity explained there is an unrestricted cyclic motion between them^10,21^.

Additionally, the experimental stimuli used here were not newly created but rather selected from mature creative ad images, which have been in the market. The electrophysiological evidence for the model of creative evaluation found in the advertising field may be extended to other actual products (e.g., creative furniture), thereby providing a possible wide and practical application of creative evaluation research results.

Our study still has some limitations, however. For example, the satiating effect may have been elicited by the repetition of the creative ads, even though the study used a random order to reduce the repletion effect. Consequently, the repletion effect may have caused reaction times of ERP analyses to be confounding variables. In fact, our experimental design focuses on exploring the evaluation of creative ads by matching relationships between the target word and ad. As such, controlling for consistency in advertising is more likely to indicate that the difference in results is due to the different target words. However, if different images are used, then the results will be affected not only by different target words but also by processing different information in the images. Nevertheless, the results will be affected by images if image processing is taken into account. Future studies should improve our experimental paradigm and adopt more efficient materials to uncover further the pure evaluation of creative ads. In addition, responses to the ads may have been influenced significantly by participant characteristics such as personality, creative abilities, and creative achievements. These variables could be investigated in future studies.

## Method

### Participants

As paid volunteers, 24 university students in China (11 females and 13 males, aged 21–26 years, mean age = 23.62 years, *SD* = 1.37) who had never been part of a similar experiment agreed to participate and signed a written informed consent. All participants were right-handed with normal or corrected vision. Using G-Power, it was calculated that, to detect a medium effect size (*f* = 0.25) with 80% power, assuming a correlation of 0.6 among repeated measures, a minimum sample size of 23 was required. In addition, this study was approved by the local ethics committee of Shanghai Normal University and was conducted in accordance with the Declaration of Helsinki (2013).

### Experimental design and stimuli

**Figure 1:**
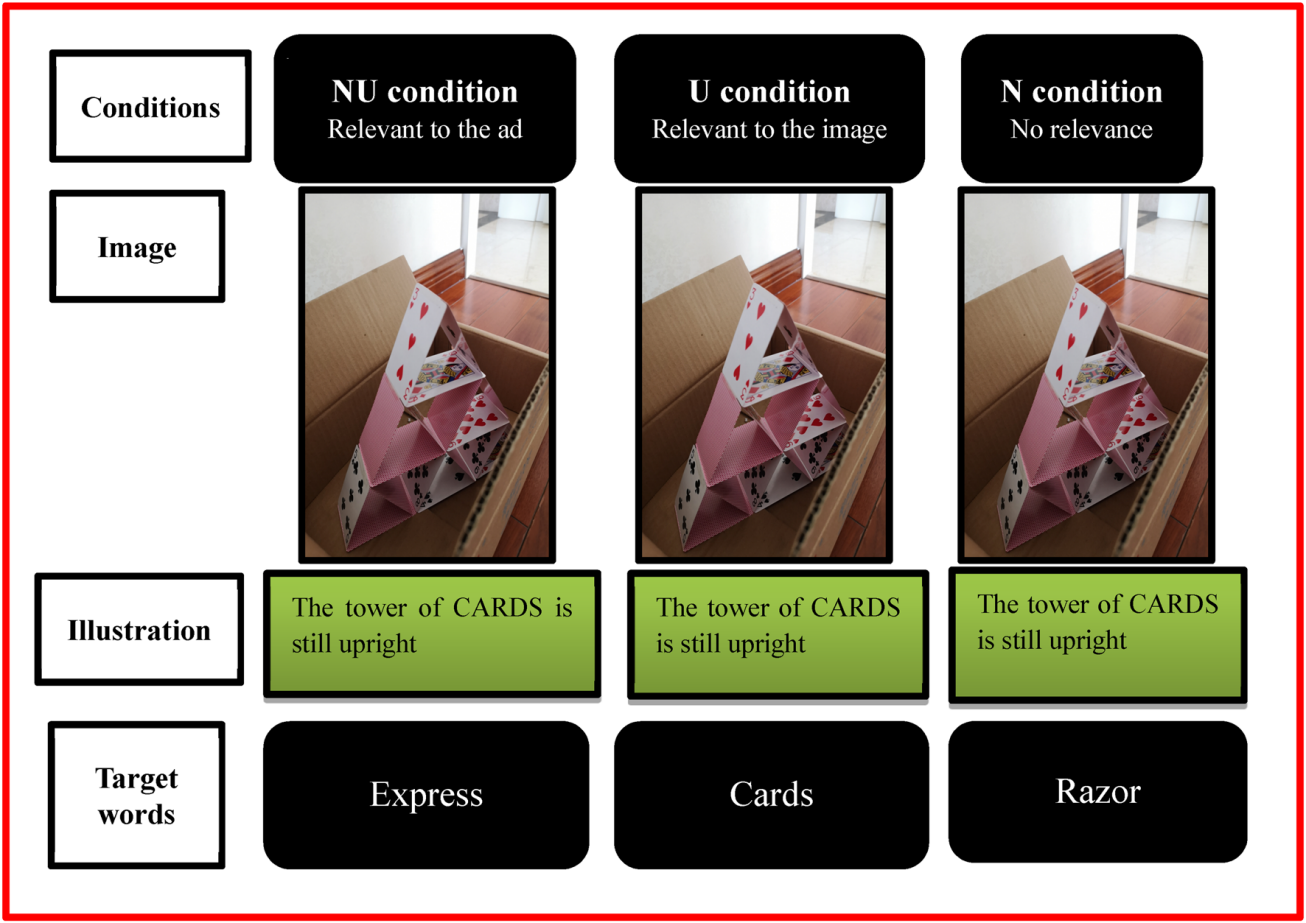
Example of experimental material under three conditions.

The materials used here were similar to that of the previous study^26^. In their study, Zhou and colleagues^26^ asked 24 participants to judge whether an ad was creative. Approximately 20 of those participants judged that each ad was creative before it was selected here. Accordingly, we collected 100 ad images that were judged to be creative. To control for the length of creative sentences, 78 ads with a similar number of words (ranging from 7 to 12 words, *M* = 9.02, *SD* = 1.56) were selected as the experimental stimuli for the present study. Subsequently, using the creative ad’s image and illustration as the basis, three experimental conditions were developed by manipulating various types of target words in Mandarin Chinese. These conditions were either the theme words of the creative ads, words relevant to the ad’s image, or irrelevant words (Fig. 1). All target words were checked for length and frequency of use in the Chinese language. No significant differences were found in word length (*M*: N = 2.95, U = 3.20, NU = 3.10; *F*(2, 124) = 1.23, *p* = 0.30, η_p_^2^ = 0.02) and word frequency (*M*: N = 117,910, U = 177,130, NU = 111,340; *F*(2, 122) = 2.83, *p* = 0.077, η_p_^2^ = 0.04) among the three conditions. The frequency in modern Chinese was computed using an online Chinese text computing resource (http://lingua.mtsu.edu/chinese-computing/). Notably, each image and illustration in the experiment would be presented in triplicate. Thus, a repetition effect would occur. To reduce this, the study conducted an entirely random treatment on the stimuli order presentation. Additionally, we limited the size of illustrations to the greatest extent possible to reduce eye movements due to long word count. As mentioned, it was unnecessary to create the target word for the NU condition because it was the theme of the creative ad. In addition, the target word for the U condition was derived from the image itself; thus, the target word would be set as the direct information presented in the image.

**Figure 2:**
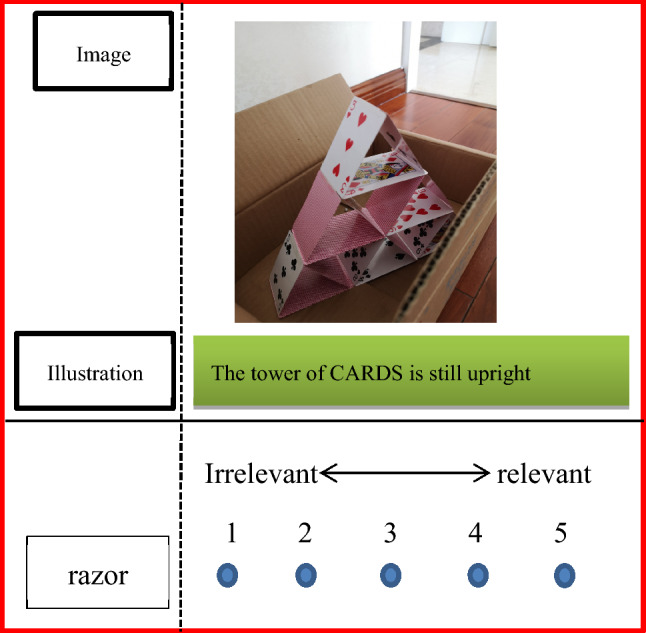
Assessment of the N condition.

For example, Fig. 1 illustrates the target word related to the image as “cards.” The target word for the N condition would relate neither to the creative theme nor information in the image. As such, the irrelevant target word depicted in Fig. 1 was proposed as “razor.” Next, the relationship between this target word and the creative ad was assessed. A total of 31 subjects (15 male and 16 female, *M* = 23.7 years, *SD* = 1.88), who did not participate in the formal experiment, rated each creative ad on a correlation scale of 1–5 through an online questionnaire (Fig. 2). A high score indicated highly relevant results. A total of 62 ads with scores close to 1 (*M* = 1.05, *SD* = 0.06) were selected from the 78 ads. Under the three conditions, the number of target words was controlled within 2–5 Chinese characters.

### Procedure

**Figure 3:**
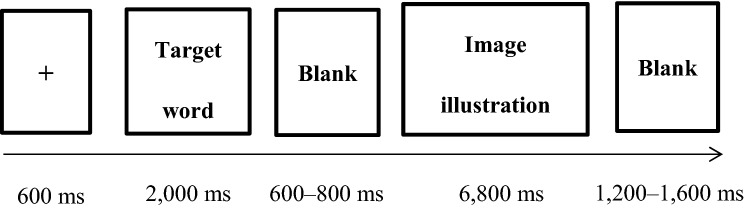
Illustration of the experimental trial.

E-prime 2.0 software was used for programming and recording reaction data. Before the experiment, the subjects were instructed to avoid blinking and making unnecessary movements as much as possible during the experiment. Each ad image had three types of target words. In all, 186 trials for 62 images were equally divided into three blocks, each consisting of 62 trials per condition. The presentation order of the trials was randomized in each block, and the sequence of the blocks was balanced across participants. As seen in Fig. 3, each trial started with a fixation “ + ” in the middle of the screen for 600 ms. Then, the target character (e.g., express) was presented for 2000 ms and the participants were instructed to remember the target word. After a jitter of 600–800 ms, an ad (image above + illustration below) was presented in the center of the screen for 6800 ms (set in accordance with the participants’ longest response time during the stimuli evaluation period). The participants were required to evaluate the relationship between the presented ad and the memorized target word. The participants pressed the “1” key if they evaluated the target word was irrelevant to the ad, the “2” key if they evaluated that the target word only described the information of the ad image, and the “3” key if they evaluated that the target word was the theme of the presented ad. A random black screen was displayed sometime between 1200 and 1600 ms in each trial. The participants could rest for approximately 30 s after every 50 trials. The entire experiment started with a certain number of exercises to familiarize the participants.

### ERP recording

Brain electrical activity was recorded using 64 Ag/AgCl electrodes that were sewn into an elastic cap (NeuroScan Inc., USA) with the reference on the left mastoid. The 10–20 extended electrode system was used for electrode position. Recordings were made on a vertical electrooculogram (with electrodes positioned above and below the right eye) and a horizontal electrooculogram (with electrodes positioned on the outer canthi of each eye). Interelectrode impedance was maintained below 5 kΩ. The electroencephalogram and electrooculogram were recorded at a sample rate of 500 Hz. A bandpass filter was set at 0.01–100 Hz. Ocular artifacts were rejected offline. Single trials were rejected when the response was improper or contaminated by blinks, eye-movements, and excessive muscle activity (voltage exceeded ± 100 in any channel).

### ERP data analysis and statistics

The ERP waveforms were time-locked to the onset of the stimuli “image + illustration.” An epoch was analyzed to be 1200 ms (including the 200 ms prior to the stimulus onset as the baseline). Only trials where the “3” key was pressed in the NU condition, the “2” key in the U condition, and the “1” key in the N condition were considered for ERP analysis.

**Figure 4:**
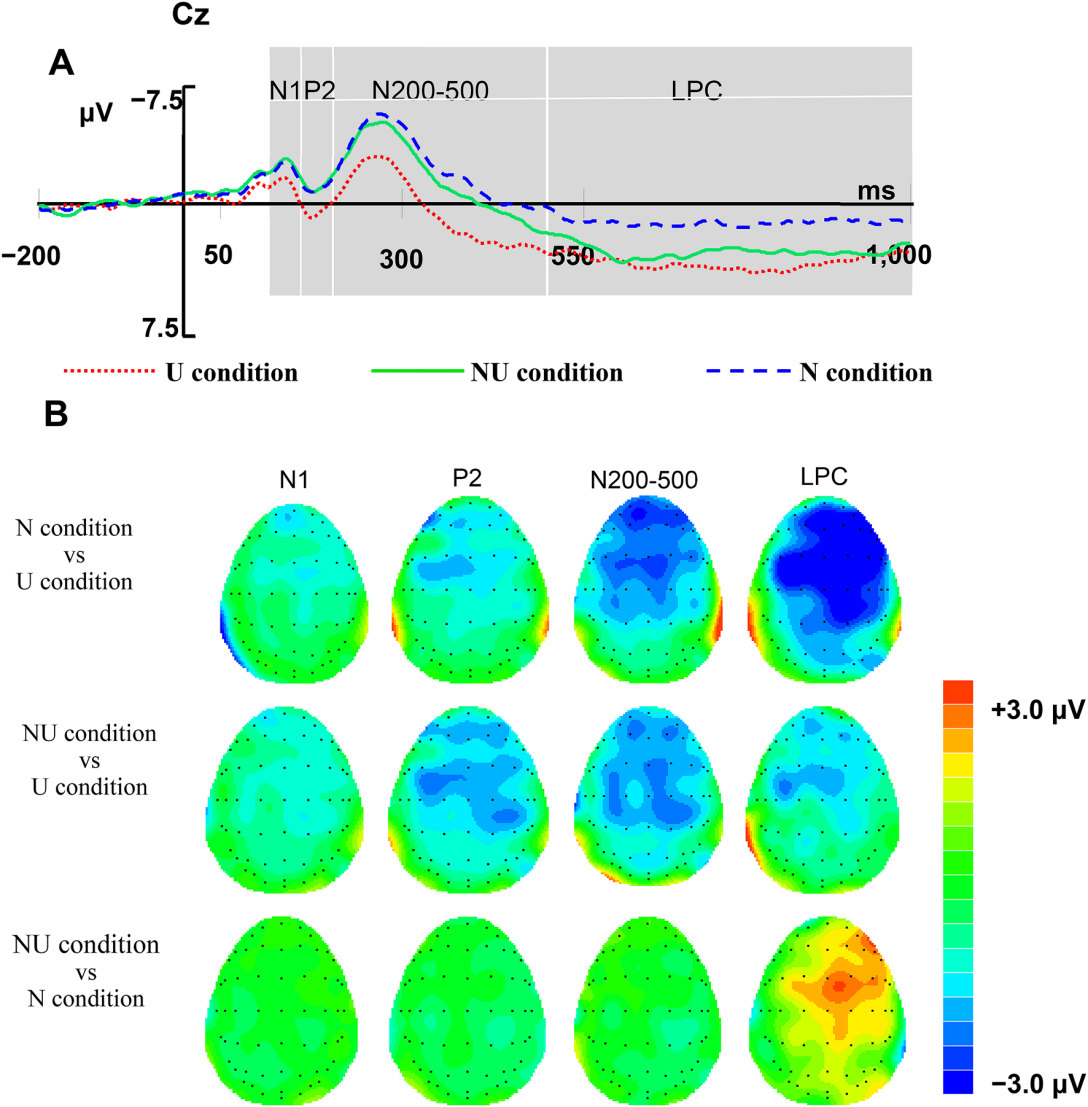
(**A**) Grand average waveform of the three conditions at Cz point. Each condition is represented by a different color (red, green, and blue for U, NU, and N, respectively). (**B**) Topographic maps of difference waves (N condition versus U condition, NU condition versus U condition, and NU condition versus N condition) within time ranges 120–160, 160–200, 200–500, and 500–1000 ms.

On the basis of the grand-averaged waveform (Fig. 4A) at approximately 120 ms, the ERP waveform induced by the two novelty conditions was separated from the waveform induced by the U condition. The separation was mainly reflected in the following components: N1 (120–160 ms), P2 (160–200 ms), and N200-500 (200–500 ms). In the 500–1,000 ms time window, the two usefulness conditions induced a more positive ERP component than the N condition. In the topography map (Fig. 4B), as the distribution described above^26,31,32,37–39^, the frontal N1 and fronto-central P2 were measured separately in the 120–160- and 160–200-ms time windows, respectively. And mean voltages in the time window of 200–500 ms and 500–1000 ms were measured at both anterior and posterior electrodes. Consequently, The following 21 sites were chosen for statistical analysis: FP1, FPZ, FP2, F3, Fz, F4, FC3, FCz, and FC4 (9 sites for frontal); C3, Cz, C4, CP3, CPz, and CP4 (6 sites for central); P3, Pz, P4, PO3, POz, and PO4 (6 sites for occipital). Accordingly, we conducted a two-factor repeated measure analysis of variance (ANOVA) for the mean amplitudes of each component. Specifically, The ANOVA factors were condition (three level: NU, N, U), and electrode site (9 frontal sites for N1, 15 frontal and central sites for P2, and 21 sites for N200-500 and LPC). SPSS 16.0 was used to perform the repeated ANOVA measures. For all analyses, a threshold significance criterion of *p* < 0.05 was used. When the factors failed to satisfy the sphericity assumption, we used the Greenhouse–Geisser correction. Post-hoc analyses were conducted using Bonferroni’s corrected *t* tests.
